# Child Rights and Child Poverty: Can the International Framework of Children's Rights Be Used to Improve Child Survival Rates?

**DOI:** 10.1371/journal.pmed.0040307

**Published:** 2007-10-23

**Authors:** Simon Pemberton, David Gordon, Shailen Nandy, Christina Pantazis, Peter Townsend

## Abstract

The authors explain how the international framework of human rights can be better used to help reduce child poverty and improve child survival rates.

The purpose of this short paper is to explain how the international framework of human rights can be better used to help reduce child poverty and improve child survival rates.

## The Consequences of Child Poverty

It is estimated that over 10 million children in developing countries die each year, mainly from preventable causes. In approximately half of these deaths, malnutrition is a contributory cause [1,2]. However, the World Health Organization has argued that seven out of ten childhood deaths in such countries can be attributed to just five main causes, or their combination. In addition to malnutrition [3], these causes are pneumonia, diarrhoea, measles, and malaria. Around the world, three of every four children seen by health services are suffering from at least one of these conditions. Many of these deaths could be prevented using readily available medical technologies at comparatively little cost. In 1997, the United Nations Development Programme estimated that the cost of providing basic health and nutrition for every person on the planet was $13 billion per year for ten years [4]. To place this sum in perspective, in 2002, the United States population spent $30 billion on pizza and Europeans spent $12 billion on dog and cat food.

While medical interventions can, in principle, prevent most young children from dying early, they cannot remove the underlying causes of poor health, which are linked directly to the severely deprived or absolutely poor living conditions suffered by 30% of the world's children [5,6]. For example, almost a third of the world's children live in squalid housing conditions, with more than five people per room or with mud flooring. Over half a billion children (27%) have no toilet facilities whatsoever and over 400 million children (19%) are drinking from unsafe open water sources (e.g., rivers, lakes, ponds) or have to walk so far to fetch water that they cannot carry enough to meet minimum health requirements [6]. The World Health Organization has argued that: “The world's biggest killer and the greatest cause of ill health and suffering across the globe is listed almost at the end of the International Classification of Diseases. It is given code Z59.5—extreme poverty” [7]. Eliminating extreme poverty is the key to improving global child survival rates, particularly over the long term.

## Child Survival and Child Rights

In recent years, the importance of the link between child rights and child survival has been contested. In 2004, an editorial in *The Lancet* [8] argued that UNICEF's focus on child rights had been detrimental to international campaigns to improve child survival. In particular, the article claimed that the outgoing UNICEF Director (Carol Bellamy) had focused on “girl's education, early childhood development, immunisation, HIV/AIDS, and protecting children from violence, abuse, exploitation, and discrimination”, and that in doing this she had “failed to address the essential health needs of children”. The current Director of UNICEF (Ann Veneman) has so far given much less prominence to child rights, making “child mortality public enemy number one for the agency” [9].

We argue that a rights-based strategy will increase child survival, in part by reducing child poverty, but only if some rights are prioritised over others. UNICEF, under Bellamy, adopted a position in which all the rights in the UN Convention on the Rights of the Child (UNCRC) were regarded as of equal importance, and both developed and developing countries were urged to realise these rights progressively (i.e., one after the other) [5,10]. This position has become hard to defend, since some rights are clearly more important than others and/or contingent on others. For example, whilst UNICEF recognises that children living in poverty are more likely to experience non-fulfilment of other rights [5], the right to vote is little use to a child who has died in infancy as a result of a lack of medical care due to poverty.

There is a clear need to prioritise the realisation of rights in policy so that action can be divided into successive stages according to degree of severity of transgression and available resources. Ensuring child survival provides a good basis for this prioritisation, but to be effective, actions need to tackle both the symptoms and the underlying causes. The UNCRC (see Box 1) established a strong ideological, moral, and political tool for challenging these structural causes and its utility should not be undervalued.

**Box 1. The Five Core Principles of the UN Convention on the Rights of the Child**

- The right to life, survival, and development
- Non-discrimination
- Devotion to the best interests of the child
- Respect for the views of the child
- The right to an adequate standard of living and social security

Article 24 (1) of the UNCRC states that:

*“States Parties recognize the right of the child to the enjoyment of the highest attainable standard of health and to facilities for the treatment of illness and rehabilitation of health. States Parties shall strive to ensure that no child is deprived of his or her right of access to such health care services”.*

Similarly, Article 24 (2) of the UNCRC continues:

*“States Parties shall pursue full implementation of this right and, in particular, shall take appropriate measures:*

*(a) To diminish infant and child mortality;*

*(b) To ensure the provision of necessary medical assistance and health care to all children with emphasis on the development of primary health care;*

*(c) To combat disease and malnutrition, including within the framework of primary health care, through, inter alia, the application of readily available technology and through the provision of adequate nutritious foods and clean drinking-water, taking into consideration the dangers and risks of environmental pollution;*

*(d) To ensure appropriate pre-natal and post-natal health care for mothers;*

*(e) To ensure that all segments of society, in particular parents and children, are informed, have access to education and are supported in the use of basic knowledge of child health and nutrition, the advantages of breastfeeding, hygiene and environmental sanitation and the prevention of accidents;*

*(f) To develop preventive health care, guidance for parents and family planning education and services.”*

If these rights were to be fulfilled, child survival rates would rapidly improve.

## The Potential of a Human Rights Approach

A human rights approach offers the possibility for progressive interventions into child poverty and child survival in three ways. First, conventions like the UNCRC have been signed by most countries in the world and thus can be considered to embody universal values and aspirations. Second, human rights conventions place a legal obligation upon states, a view endorsed by Mary Robinson (former UN High Commissioner for Human Rights) in her speech to the 2002 World Summit on Sustainable Development in Johannesburg, South Africa: “…a human rights approach adds value because it provides a normative framework of obligations that has the legal power to render governments accountable” [11].

Any comprehensive understanding of the root causes of poverty and the 10 million annual premature child deaths cannot ignore the legal and institutional structures that create and perpetuate income and wealth imbalances within society. Thus, human rights provide a challenge to these structures [12].

Third, rights-based language can help to direct policy. It shifts the focus of debate from the personal failures of the “poor” to the failure of macro-economic structures and policies implemented by nation states and international bodies (World Trade Organization, World Bank, International Monetary Fund, etc.) to eradicate poverty. Hence, child poverty in this context is no longer described as a “social problem” but a “violation of rights” [13].

## Human Rights as a Tool for Poverty Reduction: Some Practical Issues

There are objections to the human rights approach. One question is whether human rights, as formally expressed in human rights conventions, are genuinely universal [14]. Critiques based on cultural relativism and Asian values have suggested that human rights are “western” in orientation and content and, consequently, promote liberal/individualist social preferences over more “collective” forms of organisation [15,16]. However, it is a fact that every country in the world (the 193 UN Member States) has signed the UNCRC—implying that negotiated moves towards the realisation of the agreed goals are feasible. There is a near-unanimous consensus on objectives and values. Only two countries have to date failed to ratify the UNCRC—Somalia and the US.

A second question is whether economic, social, and cultural rights (including child health and survival) have been subjugated to civil and political rights, despite the insistence of human rights advocates on the “indivisibility” of these rights (see Box 2 for definitions of different categories of rights) [17]. Following the Universal Declaration of Human Rights in 1948, civil and political rights have tended to be promoted over economic, social, and cultural rights [18]. Two specific international covenants were agreed upon: the International Covenant on Civil and Political Rights and the International Covenant on Economic, Social and Cultural Rights, and signatories are committed to the realisation of all these rights [18,19]. Ironically, the act of creating two covenants has served to provide contradictory messages about the “indivisibility” of rights. This distinction has become entrenched in the legal systems of nation states, which sometimes place civil and political rights in the “justiciable” section of their constitution, while relegating economic, social, and cultural rights to the realm of directive principles [20]. Civil and political rights have entered into law ahead of economic, social, and cultural rights, which are crucial for poverty eradication and health improvements.

**Box 2. Definitions of Categories of Rights**

*Social and economic rights* relate to guaranteeing individuals a minimum standard of living, such as a minimum income, housing, health care, and education.

*Cultural rights* relate to the recognition and safeguarding of ethnic/religious groups' practices and beliefs.

*Civil rights* relate to personal freedoms, such as the right to privacy, freedom of movement, and right to a fair trial.

*Political rights* relate to political participation, such as the right to vote and the right to peaceful assembly.

A third question about human rights is whether the “non-justiciability” and non-enforcement of certain economic, social, and cultural rights makes the development of anti-poverty policies difficult. It is often argued that “rights”, as they have been defined in human rights conventions, are imprecise or are moral claims that are not legally enforceable [20]. Many “rights” have so far been largely ignored by national courts, and the realisation of economic, social, and cultural rights is particularly difficult. Domestic courts have been adept at arriving at complex decisions in cases relating to civil and political rights, but they have tended to dodge issues of poverty, access to health care, and non-fulfilment of other economic and social rights. They cite the non-justiciability of such rights and have not been aided by international jurisprudence, which is currently lacking in this area.

However, both domestic and international judiciaries could follow the inventive and progressive approach of treaty committees and special rapporteurs who scrutinise and regularly report on nation states' adherence to the conventions [20]. For instance, the Committee on the Rights of the Child has, on a number of occasions, refused to accept the “non-affordability” claims made in the progress reports of states. For instance, in the light of the funding of their defence budgets, Indonesia and Egypt were invited to justify their failure to make significant progress in implementing the UNCRC [20].

There are notable examples where economic and social rights have been written into nation states' constitutions. Rights thus removed from the political sphere into the legal sphere are less contested. The advantage of this shift is that the courts can help to set minimum welfare standards—through reviewing government budgets, vetoing legislation that is likely to increase rather than reduce poverty, and so on. Examples of such an approach can be found in India, the Republic of South Africa, and Finland [21].

## The Relationship between the Rights of the Child and Child Poverty

The UNCRC does not contain an explicit human right to freedom from poverty. Hence, to measure poverty in terms of rights, a selection process is required to match these rights to the severe deprivations of basic human need that characterise poverty and cause ill health. Giving greater priority to selected groups of rights does not imply that rights are divisible in any ultimate or “perfect” sense. It allows planned actions to be taken, progressively by stages, to achieve agreed ends. Human rights are interrelated, so the fulfilment of some rights is reliant on the prior realisation of others [15].

Many of the rights, as expressed in the relevant charters and conventions, are ambiguous or imprecise. This is often the case with social and economic rights where access to some rights is easier to define and measure than others. The right to survival—preventing early deaths—is less difficult to measure than access to adequate health or educational services. Many phenomena (such as “health”) can be considered to be on a continuum ranging from “good health” to “poor health/death” [22]. Similarly, fulfilment of rights can be considered to be on a continuum ranging from complete fulfilment to extreme violation. Courts can make judgments on individual cases on the correct threshold level at which rights are found to have been violated or fulfilled (see Figure 1).

**Figure 1:**
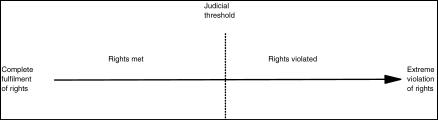
Continuum of Rights

Regrettably, there is little international case law at present that identifies the location of this “judicial” threshold with respect to many social, economic, and cultural rights, such as the right to health care. Social scientists therefore have a responsibility to help identify such “judicial” thresholds—a methodological issue we have sought to address in previous research [23].

## Conclusion

The international framework of child rights is a useful theoretical and political tool in taking action to reduce child poverty and improve child health [24–29]. A rights-based strategy is necessary to the development not only of international and national jurisprudence but to a global civil society that challenges the structures of global poverty, so that child rights may move from the realms of rhetoric to those of tangible reality. However, in order to provide clear guidance for policy, we need to move away from an approach that gives all rights equal weight, to a strategy of choosing clear implementation priorities. We suggest that the rights contained in the UNCRC relating to child survival and non-discrimination be prioritised, i.e., these rights should be implemented first in situations where child rights cannot be implemented all at once. An emphasis on both survival and non-discrimination is vital to prevent unequal health provision from developing—for example, privileging the survival of boys over girls or one ethnic group over another. If such priorities are not set, then governments may decide to implement those rights first that are least expensive and easiest to fulfil and only implement more expensive rights, which would improve child survival, at a later date.

Child rights fulfilment by states can only be properly assessed within the global context of poverty and an equal appraisal of developed and developing countries. Thus, the guidance given by the Committee on the Rights of the Child (General Comment No. 5) [30], which specifies that the realisation of child rights is the responsibility of *all* nation states, be it within their jurisdiction or through international cooperation and action, requires widespread reinforcement and support. This places special obligations upon those who operate in the interests of the powerful nation states at the supranational level to ensure that child survival rates are improved by the fulfilment of children's human rights, particularly their economic and social rights. Solely concentrating on medical interventions that increase child survival, while ignoring other violations of children's human rights, is unlikely to ensure the health and well being of children in the long term.

## Abbreviations

UNCRC: United Nations Convention on the Rights of the Child
